# King Edward's Fund and the Evelina Hospital

**Published:** 1912-02-24

**Authors:** D. Malcolm Scott

**Affiliations:** Chairman of Committee of Management. Evelina Hospital, Southwark Bridge Road


*To the Editor of* The Hospital.

### KING EDWARD'S FUND AND THE EVELINA HOSPITAL.

*To the Editor of* The Hospital.

Sir,—In justice to my Committee I trust you will find space for this reply to the letter from the Honorary Secretaries of King Edward's Hospital Fund, which you have published in your issue of February 17.

Their communication will, I fear, give the impression that my Committee have deliberately departed from the Uniform System of Hospital Accounts; this is not the case. It must be remembered that the legacy in question was absolutely unconditional, and *not* specified as being for the general purposes of the hospital. My Committee, however, as a precaution sought legal advice, and ascertained that it would be a perfectly legitimate transaction to allocate it to the Samaritan Fund. This legacy was then dealt with in accordance with a regulation of the King's Fund (No. 7 on page 12 of their Red Book) which exactly applies to the case. This course has been approved by the hospital auditor, who signed the accounts.

Paragraph 3 of the Honorary Secretary's letter states that my Committee have full power to apply the legacy to the general purposes of the hospital. Granted, but having regard to the conditions just mentioned, they have also equal power to allocate it to any other object which is for the benefit of the hospital.

If the Committee of the King's Fund would be just in dealing with this matter, they must admit that it is not merely a question of hospital accounts, but also the strict interpretation of the wording of a will.

The letter from the Honorary Secretaries cannot fail to create the impression that my Committee by their action wish to conceal the purposes for which the income of the Samaritan Fund is used. To this I must give a complete denial, and would point out that the authorities of the King's Fund know full well that the accounts of all special funds are published in our Annual Report, and are therefore open to the inspection and criticism not only of the subscribers but of anyone who may obtain a copy of the book.

In managing the affairs of this institution, which is the only large hospital for children in the whole of South London, my Committee's only desire is to do, with the means at their disposal, the greatest amount of good for the sick poor, and by their straightforward policy hope to elicit the sympathy and support of the public.—Yours faithfully,

Evelina Hospital, D. Malcolm Scott,

Southwark Bridge Road, Chairman of Com-

February 17. mittee of Management.

